# Identification of Lung and Blood Microbiota Implicated in COVID-19 Prognosis

**DOI:** 10.3390/cells10061452

**Published:** 2021-06-10

**Authors:** Kypros Dereschuk, Lauren Apostol, Ishan Ranjan, Jaideep Chakladar, Wei Tse Li, Mahadevan Rajasekaran, Eric Y. Chang, Weg M. Ongkeko

**Affiliations:** 1 Division of Otolaryngology-Head and Neck Surgery, Department of Surgery, University of California, San Diego, CA 92093, USA; kderesch@ucsd.edu (K.D.); laaposto@ucsd.edu (L.A.); iranjan@ucsd.edu (I.R.); jchaklad@ucsd.edu (J.C.); wtl008@ucsd.edu (W.T.L.); 2 Research Service, VA San Diego Healthcare System, San Diego, CA 92161, USA; 3 Department of Urology, University of California San Diego, La Jolla, CA 92093, USA; mrajasekaran@health.ucsd.edu; 4 Urology Service, VA San Diego Healthcare System, San Diego, CA 92161, USA; 5 Department of Radiology, University of California, San Diego, CA 92093, USA; e8chang@health.ucsd.edu; 6 Radiology Service, VA San Diego Healthcare System, San Diego, CA 92161, USA

**Keywords:** microbiome, lung microbiota, blood microbiota, SARS-CoV-2, COVID-19, coronavirus, inflammation

## Abstract

The implications of the microbiome on Coronavirus disease 2019 (COVID-19) prognosis has not been thoroughly studied. In this study we aimed to characterize the lung and blood microbiome and their implication on COVID-19 prognosis through analysis of peripheral blood mononuclear cell (PBMC) samples, lung biopsy samples, and bronchoalveolar lavage fluid (BALF) samples. In all three tissue types, we found panels of microbes differentially abundant between COVID-19 and normal samples correlated to immune dysregulation and upregulation of inflammatory pathways, including key cytokine pathways such as interleukin (IL)-2, 3, 5-10 and 23 signaling pathways and downregulation of anti-inflammatory pathways including IL-4 signaling. In the PBMC samples, six microbes were correlated with worse COVID-19 severity, and one microbe was correlated with improved COVID-19 severity. Collectively, our findings contribute to the understanding of the human microbiome and suggest interplay between our identified microbes and key inflammatory pathways which may be leveraged in the development of immune therapies for treating COVID-19 patients.

## 1. Introduction

As of 19 March 2021, 122,044,376 people have been infected and 2,695,014 have died from coronavirus disease 2019 (COVID-19), caused by the severe acute respiratory syndrome coronavirus 2 (SARS-CoV-2) [1]. Symptoms and severity of COVID-19 vary drastically. The most common symptoms are fever, cough, fatigue, headache, myalgias, and diarrhea. The severity of COVID-19 symptoms can range from very mild to severe [2,3]. Around one in six infected individuals present with no symptoms at all [4]. Approximately 10% of people infected with COVID-19 experience symptoms’ that persist beyond three weeks in what is known as “long haul COVID-19” [5]. The cause of the large variance in COVID-19 severity and length is not fully understood, and research into this topic is of great importance to learn how to prevent long haul COVID-19 [6,7]. In more severe cases of COVID-19, the innate immune system fails to stop viral replication of SARS-CoV-2, leading to a substantial immune response from immune effector cells which has previously been characterized by a large amount of cytokines in the body. This hyperinflammatory condition manifested as a ‘cytokine storm’ is called COVID-19 ARDS, and this is one of the most dangerous and potentially life-threatening events related to COVID-19 [8].

The gut microbiome and lung microbiome have both been found previously to be altered in COVID-19 patients, in addition to likely playing a role in explaining the variation in critical COVID-19 response [9,10,11]. The lung microbiome has been shown to play an important role in maintaining lung homeostasis and plays a role in prompting an adequate immune response to pathogens, preventing a hyper-inflammatory response via crosstalk between microbes and receptors on immune cells within the lungs, while some microbes/microbiota compositions appear to prompt more inflammatory responses to pathogens [11,12,13,14]. Previous study has found that severity of COVID-19 disease is correlated with the predominance of opportunistic pathogens in the gut [10]. Building upon this prior research, we wished to investigate the microbiome further by correlating microbe abundance, both fungi and bacteria, to COVID-19 severity, immune cell abundances, and inflammatory and immune-associated gene pathways, and investigate, in addition to the lung microbiome, the effect the blood microbiome may have in modulating the immune response to COVID-19. Thus, in this study we aimed to characterize the lung and blood microbiome and their implication on COVID-19 prognosis through analysis of peripheral blood mononuclear cell (PBMC) samples, lung biopsy samples, and bronchoalveolar lavage fluid (BALF) samples.

## 2. Materials and Methods

**Figure 1:**
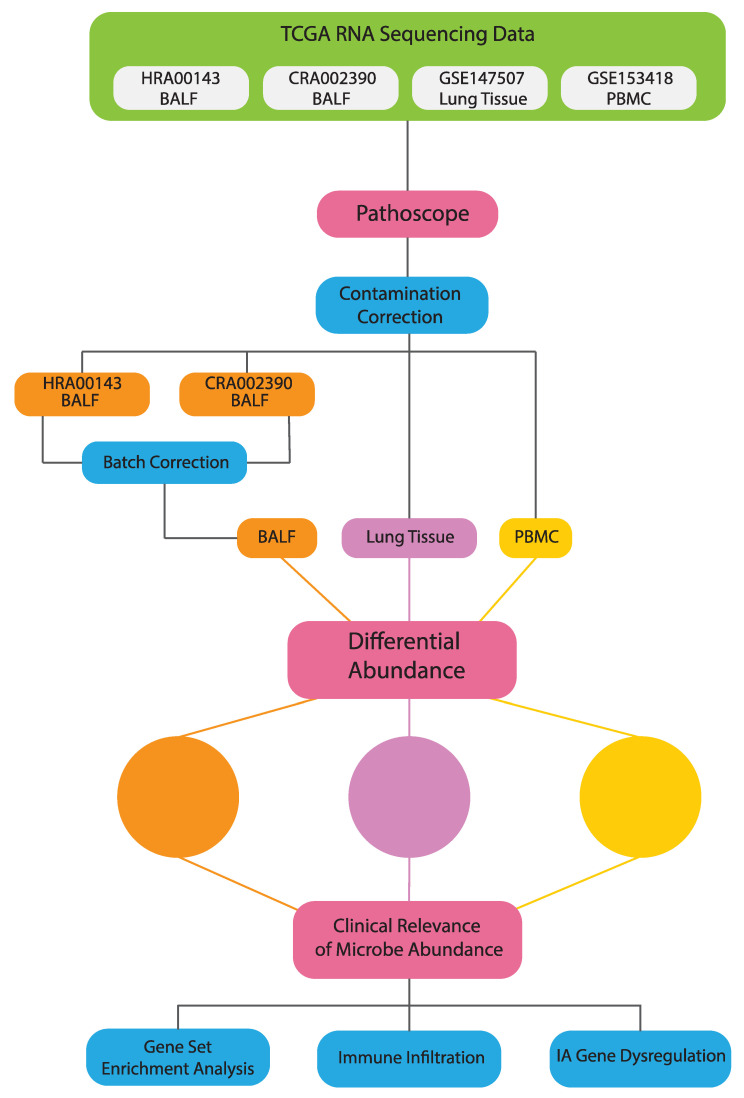
Schematic of analysis and workflow.

An overview of our methods is presented in Figure 1.

### 2.1. Data Acquisition

RNA-seq data of 17 PBMC normal samples and 17 PBMC COVID-19 samples were downloaded from GEO (accession code GSE152418 https://www.ncbi.nlm.nih.gov/geo/ accessed 15 July 2020), 8 lung biopsy normal samples and 8 lung biopsy COVID-19 samples were downloaded from GEO (accession code GSE147507 https://www.ncbi.nlm.nih.gov/geo/ accessed 15 July 2020), and 20 BALF normal samples and 8 BALF COVID-19 samples were downloaded from GSA (accession code HRA000143 https://bigd.big.ac.cn/gsa-human/ accessed 15 July 2020) and 4 additional BALF COVID-19 samples were downloaded from GSA (accession code CRA002390 https://bigd.big.ac.cn/gsa-human/ accessed 15 July 2020). Arunachalam et al. determined disease severity for PBMC samples and classified patients as convalescent, moderate, severe, or ICU [15]. The dataset included 1 convalescent sample, 4 moderate samples, 8 severe samples, and 4 ICU samples.

### 2.2. Extraction of Microbial Reads

Pathoscope 2.0 (Boston University School of Medicine, Boston, MA, USA) was used to separate the microbe-specific reads incorporated in the human reads of high-throughput RNA-seq and align it to the reads in a target library, producing levels of microbe abundance and individual taxonomic lineage [16]. This was done two times, once with a target library containing reads of bacteria and once with a target library containing reads of fungus. Microbes with total abundance less than the number of COVID-19 patients per tissue type were excluded from analysis. The batch correction method ComBat was used to adjust for batch effects when combining BALF data from the HRA000143 and CRA002390 datasets [17].

### 2.3. Differential Microbial Abundance between COVID19 and Normal Patients

The Kruskal-Wallis statistical test was used to determine differential abundance between COVID-19 samples and normal samples and correlations between microbe abundance and disease severity (*p* < 0.05). For correlation between microbe abundance and disease severity, ICU and Severe patients were grouped together into one group called Severe.

### 2.4. Correlation between Microbial Abundance and IA Gene Expression

All RNA-seq samples were aligned to human genome version GRCh38.p13 and its annotation from NCBI using STAR (Spliced Transcript Alignment to a Reference) (Cold Spring Harbor Laboratory, Cold Spring Harbor, New York, NY, USA) version 2.7.4 with sjdbOverhang set to optimize value of 99. Other parameters were left to default values [18]. We used feature counts function from package Rsubread v2.0.1 (Subread Sequence Alignment and Counting for R) (Olivia Newton-John Cancer Research Institute, Melbourne, Australia) to obtain raw read count and the result was subsequently passed to EdgeR v3.28.1 (Walter and Eliza Hall Institute of Medical Research, Parkville, Australia) for normalization using TMM metrics. TMM normalization enables us to compare gene expression across samples. The Kruskal-Wallis statistical test was used to correlate gene expression with COVID-19 status and to correlate microbe abundance to dysregulated immune associated (IA) genes.

### 2.5. Correlation between Microbial Abundance and Immune Cell Abundances

The CIBERSORTx software (Stanford University, Stanford, CA, USA) was used to deconvolute RNA-sequencing data to estimate the infiltration levels of 22 immune cell types. These immune cell types include the following: CD8 T-cells, CD4 naïve T-cells, CD4 memory resting T-cells, CD4 memory activated T-cells, follicular helper T-cells, regulatory T-cells, gamma-delta T-cells, naïve B-cells, memory B-cells, plasma cells, M0-M2 macrophages, resting dendritic cells, activated dendritic cells, resting NK cells, activated NK cells, monocytes, resting mast cells, activated mast cells, eosinophils, and neutrophils [19]. We then correlated microbe abundance with expression levels of the different immune cells using the Kruskal-Wallis statistical test. (*p* < 0.05). Patients with lower or higher microbial read counts than the median microbial read count of a particular microbe across all patients were defined as “LOW” or “HIGH,” respectively.

### 2.6. GSEA (Correlation of Microbial Abundance and Covid Status to Canonical Pathways and Immune-Associated Signatures)

We used Gene Set Enrichment Analysis (GSEA) version 4.1.0 (UCSD and Broad Institute, San Diego and Cambridge, United States) to find gene enrichment related to microbe abundance and covid status. Final best hit read numbers, scores that are used by Pathoscope to represent numbers of microbe reads in RNA-seq numbers, are used as numerical phenotype input. In separate runs, covid status was used as categorical phenotype input. We manually selected immune related pathways from CP (canonical pathways) and pooled the gene sets with C7 (immunologic signature gene sets) for gene set input. We set the number of permutations to be 1000 and no collapse gene symbols. Metric for ranking genes was set to Pearson. Everything else was left with default parameters. Significantly enriched signatures were identified by a nominal *p*-value < 0.05 and ranked by normalized enrichment score [20]. Genes involved in the three immunological signatures correlated to the greatest number of differentially abundant microbes were input into Cytoscape Reactome FIViz software version 3.7.2 to visualize the gene networks [21].

### 2.7. Contamination Correction Using Spearman’s Correlation

The abundance of individual microbes in each patient were plotted against total microbe reads in the same patient separated by tissue type to determine if any microbe is a likely contaminant. Best hit results from Pathoscope were used. If a scatterplot shows a positive slope, it suggests that the microbe was biologically relevant. If there is a vertical or near vertical slope and the counts of all the microbes are substantially above zero, then the microbe is likely a contaminant. If there is a slope close to zero or less than zero, the test is inconclusive. This reasoning follows from the assumption that similar amounts of microbes will be present regardless of how many microbes are present in the tissue sample if the microbe is an environmental contaminant.

## 3. Results

### 3.1. Differential Microbial Abundance in COVID-19 Tissue and Normal Tissue

**Figure 2:**
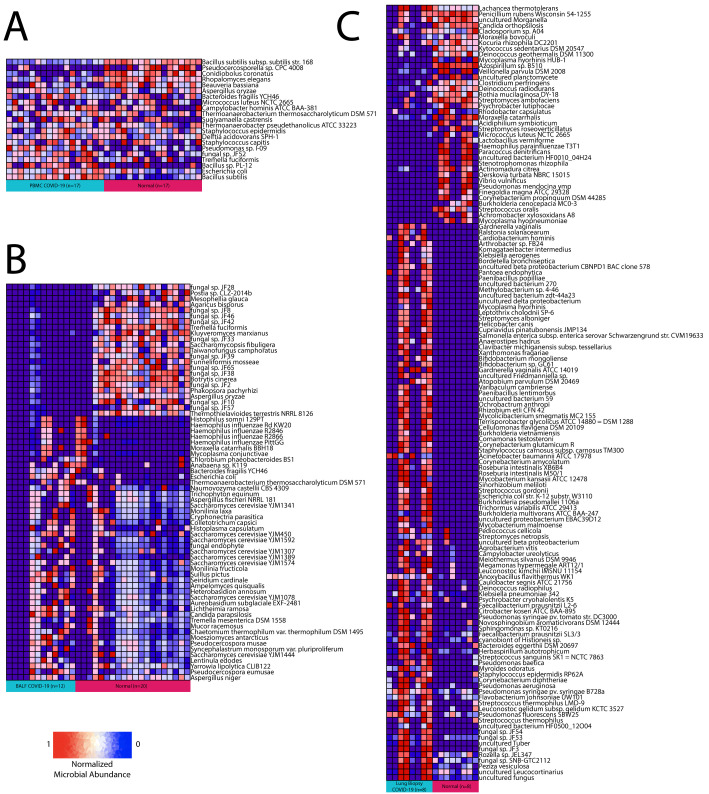
Differential abundance summary. Heatmaps showing normalized microbe abundance for COVID-19 samples vs. normal samples for (**A**) PBMC, (**B**) BALF, and (**C**) lung biopsy.

Using Pathoscope 2.0, we found 91 bacteria and 14 fungus differentially abundant in lung biopsy samples, 13 bacteria and 9 fungus differentially abundant in PBMC samples, and 12 bacteria and 57 fungus differentially abundant in BALF samples (Figure 2A–C). *Bacteroides fragilis*, *Thermoanaerobacterium thermosaccharolyticum DSM 571*, and *Escherichia* (*E.*) *coli* were the only bacteria and *Tremella fuciformis* and *Aspergillus oryzae* were the only fungus found differentially abundant between COVID-19 samples and normal samples in both BALF and PBMC tissues. There was no overlap in microbes found to be differentially abundant in lung biopsy samples and BALF or PBMC samples.

### 3.2. Immune Landscape of COVID-19 Patients

**Figure 3:**
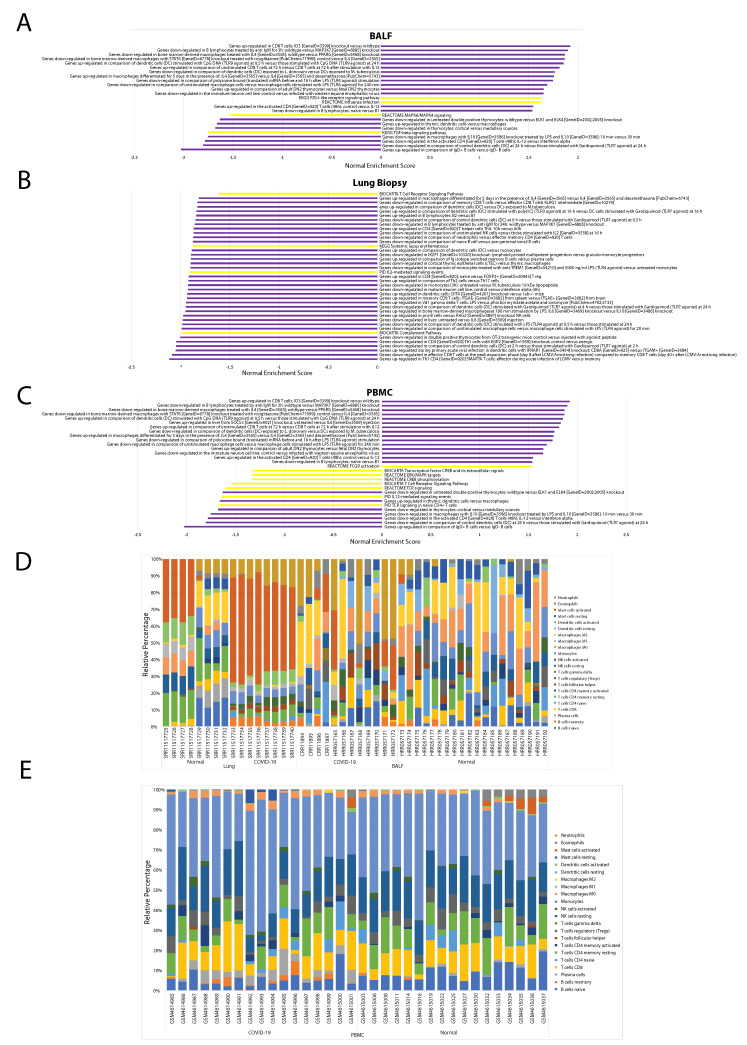
Immune landscape background. Bar plots showing nominal enrichment score for GSEA pathways significantly correlated to COVID-19 status for (**A**) BALF, (**B**) lung biopsy, and (**C**) PBMC samples. Stacked bar plots showing relative immune cell abundances for (**D**) BALF, lung biopsy, and (**E**) PBMC samples.

Most immunologic gene signatures correlated to COVID-19 status are of cells in both the innate and adaptive immune systems such as macrophages, dendritic cells, mast cells, T-cells, B-cells, and monocytes. Other results of significance include the following: across PBMC samples, T cell receptor (TCR) signaling was downregulated in COVID-19 patients, immunological signatures characteristic of increased levels of pro-inflammatory interleukins, such as IL-6, IL-8, and IL-12 were upregulated in COVID-19 patients and anti-inflammatory interleukins, including IL-4 and IL-10, were downregulated in COVID-19 patients (Figure 3C). We found plasma cells and memory B cells to be significantly increased in COVID-19 patients and naive B cells, resting memory CD4 T cells, resting natural killer (NK) cells, and Eosinophils significantly decreased in COVID-19 patients (Figure 3E).

Across BALF samples, retinoic acid-inducible gene-I (RIG-I)-like receptor signaling, transforming growth factor (TGF) β signaling, and TCR signaling were upregulated. Similar interleukin dysregulation found in PBMC samples was found in BALF samples (Figure 3A). We found activated mast cells, M1 macrophages, eosinophils, neutrophils abundances were significantly increased in COVID-19 patients and T regulatory cells (Tregs) M0 macrophages and activated dendritic cells abundance was significantly decreased in COVID-19 patients (Figure 3D).

Across lung biopsy samples, IL-4 and IL-10 signaling, TCR signaling, and the complement system pathway of the innate immune system were down regulated (Figure 3B). We found follicular helper T cells, gamma delta T cells, naive CD4 T cells, activated NK cells, and memory B cells significantly increased in COVID-19 patients and resting NK cells, plasma cells, M0, M1, and M2 macrophages, memory CD4 T cells, CD8 T cells, naive B cells, activated dendritic cells, and eosinophils significantly decreased in COVID-19 patients (Figure 3D).

### 3.3. Microbes in Blood Are Correlated to Disease Severity

**Figure 4:**
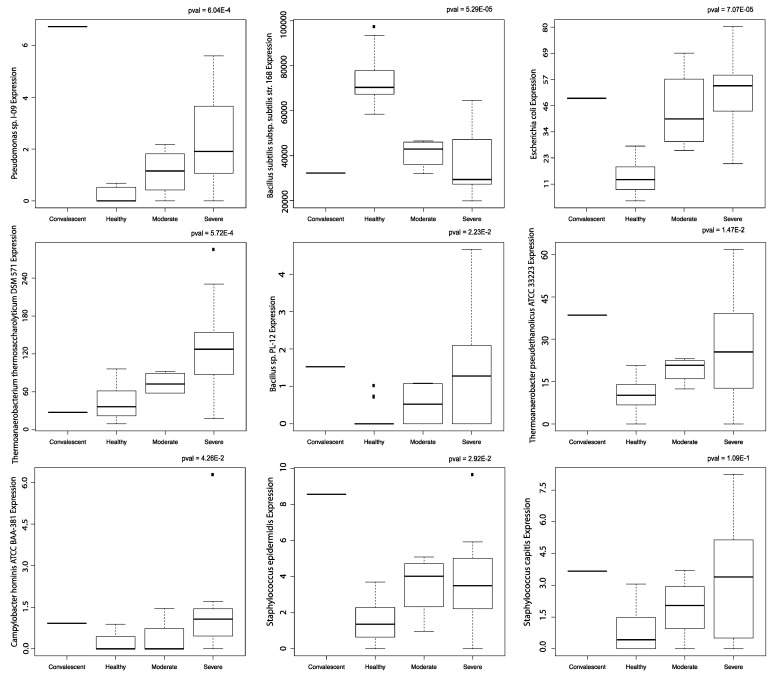
Correlation to disease severity. Boxplots of microbes significantly correlated to disease severity in PBMC samples. All boxplots were produced using the Kruskal-Wallis test.

*E. coli* abundance, *Bacillus* sp. *PL-12* abundance, *Campylobacter hominis ATCC BAA-381* abundance, *Pseudomonas* sp. *I-09* abundance, *Thermoanaerobacter pseudethanolicus ATCC 33223* abundance, *Thermoanaerobacterium thermosaccharolyticum DSM 571* abundance, and *Staphylococcus epidermis* abundance were correlated with COVID-19 severity. *Bacillus subtilis* subsp. *subtilis* str. *168* abundance was inversely correlated with COVID-19 severity (Figure 4). *Staphylococcus capitis* abundance correlation to COVID-19 severity was not significant. No significant correlation to age was found with these microbes.

### 3.4. Microbes from PBMC Samples Correlate to Immune Infiltration and to Dysregulation of Immunological Signatures and Canonical Pathways

**Figure 5:**
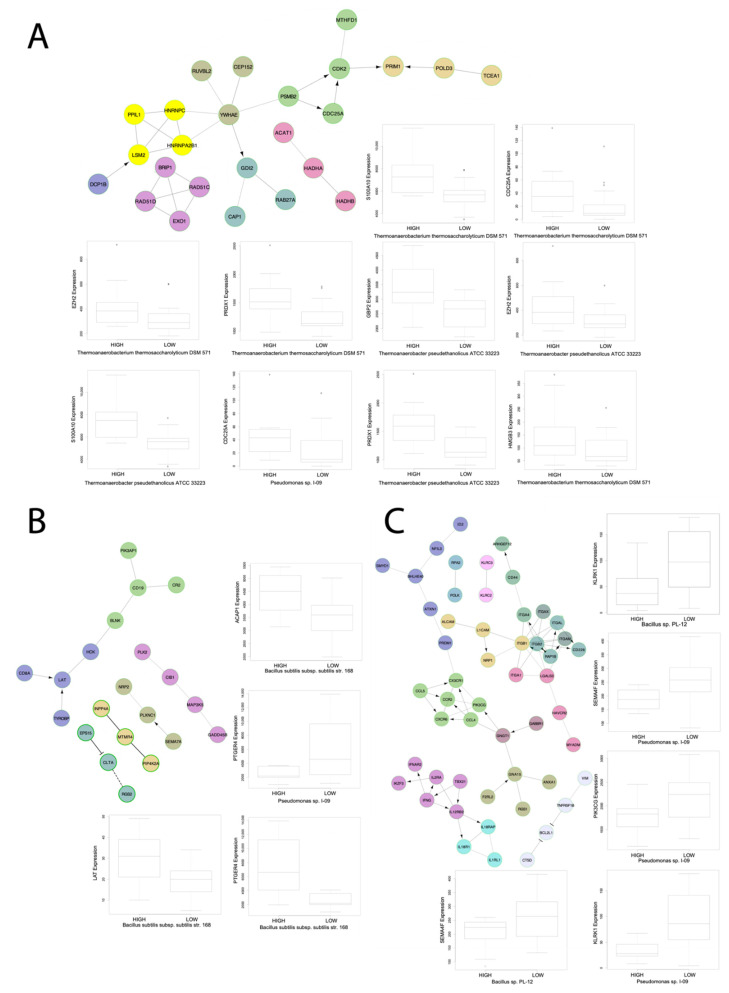
Select gene networks. Gene networks of GSEA pathways each correlated to three microbes with boxplots of microbe abundance vs. IA gene expression for microbes significantly correlated to IA gene expression for genes found to be enriched within the respective gene network. The gene networks were produced using the Cytoscape Reactome FI software. All boxplots were produced using the Kruskal-Wallis test. The networks are for the immunological signatures (**A**) GSE17974_CTRL_VS_ACT_IL4_AND_ANTI_IL12_72H_CD4_TCELL_DN, (**B**) GSE4590_SMALL_VS_LARGE_PRE_BCELL_UP, and (**C**) GSE3565_DUSP1_VS_WT_SPLENOCYTES_UP.

**Figure 6:**
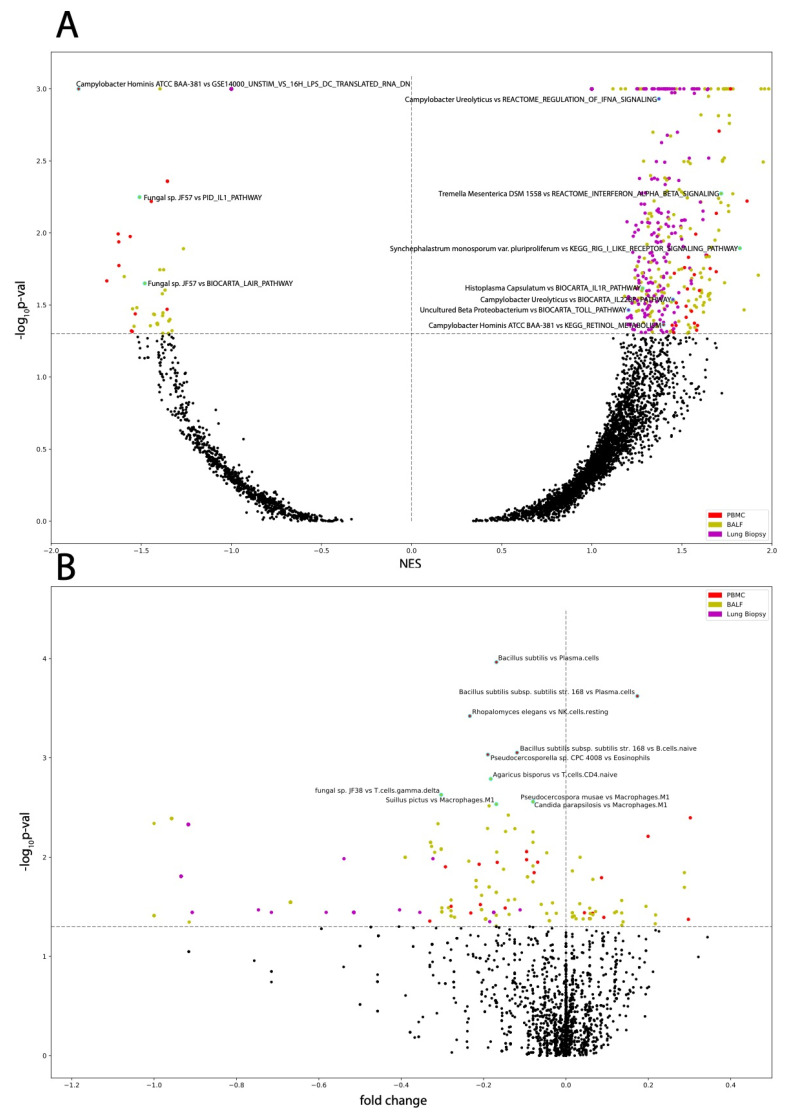
Microbe correlation to GSEA pathways and immune cells. Volcano plots of (**A**) microbe abundance correlated to GSEA pathways with the most significant pathways indicated and of (**B**) microbe abundance correlated to immune cell abundances with the most significant microbe immune cell pairs indicated. Labeled data points correspond to those with cyan outlines.

Across PBMC samples, all microbes correlated to disease severity also correlated to immune dysregulation and pathways involved in inflammation. *Bacillus subtilis* subsp. *subtilis* str. *168* was correlated to upregulation of TCR signaling, the ERK pathway, and the antigen dependent B cell activation pathway; *Bacillus* sp. *PL-12*, *Campylobacter hominis ATCC BAA-381*, *Thermoanaerobacterium thermosaccharolyticum DSM 571*, and *Thermoanerobacter pseudethanolicus ATCC 33223* all correlated to upregulation of pathways involved in increasing inflammation. *Campylobacter hominis ATCC BAA-381* and *Thermoanaerobacterium thermosaccharolyticum DSM 571* both correlated to upregulation of retinol metabolism and retinoic acid biosynthesis, which both play key roles in regulating antiviral immune response [22,23]. *Staphylococcus epidermis* correlated to dysregulation of various immunological signatures but interestingly not to any interleukin signaling pathways nor any immune cell abundances. Gene pathways for immunological signatures each correlated to the greatest number of dysregulated microbes in PBMC samples are visualized (Figure 5). *Bacillus* sp. *PL-12* and *Thermoanerobacter pseudethanolicus ATCC 33223* both correlated to upregulation of the aurora A pathway and *Thermoanerobacter pseudethanolicus ATCC 33223* correlated to upregulation of the KEGG Asthma pathway (Figure 6A,B).

### 3.5. Microbes from BALF Samples Correlate to Immune Infiltration and to Dysregulation of Immunological Signatures and Canonical Pathways

Across BALF samples, panels of GSEA and immune cell abundance correlated microbes were discovered. The most notable results include that *Saccharomyces cerevisiae YJM1444*, *Syncephalastrum monosporum* var. *Pluriproliferum*, *Candida parapsilosis*, and *Histoplasma capsulatum* correlated to upregulation of TCR signaling, inflammatory interleukin signaling, TGFβ signaling, interferon IFN α and IFNβ signaling, RIG-I-like receptor signaling, and to dysregulation of immunological signatures (Figure 6A,B).

### 3.6. Microbes from Lung Biopsy Samples Correlate to Immune Infiltration and to Dysregulation of Immunological Signatures and Canonical Pathways

Across lung biopsy samples, panels of GSEA and immune cell abundance correlated microbes were identified. The most notable results include that *Campylobacter ureolyticus*, *fungal* sp. *JF54*, *Ochrobactrum anthropi*, and *uncultured beta proteobacterium* correlated to upregulation of TCR activation, IFNα signaling, activation of the complement system, tumor necrosis factor signaling, the P38MAPK pathway, the Toll like receptor (TLR) TLR1:TLR2 cascade, and to upregulation of inflammatory interleukin signaling pathways. Additionally, *Ochrobactrum anthropi* and *uncultured beta proteobacterium* were both found inversely correlated to gamma-delta T-cell abundance. *Streptococcus sanguinis SK1* = *NCTC 7863* also correlated to upregulation of inflammatory interleukin signaling, T cell receptor signaling, and TLR signaling, and it correlated down regulation of IL-4 signaling. Other *Streptococcus* species did not correlate to interleukin signaling or inflammatory pathways (Figure 6A,B).

### 3.7. Negligible Contaminants Found in Differentially Abundant Microbes

**Figure 7:**
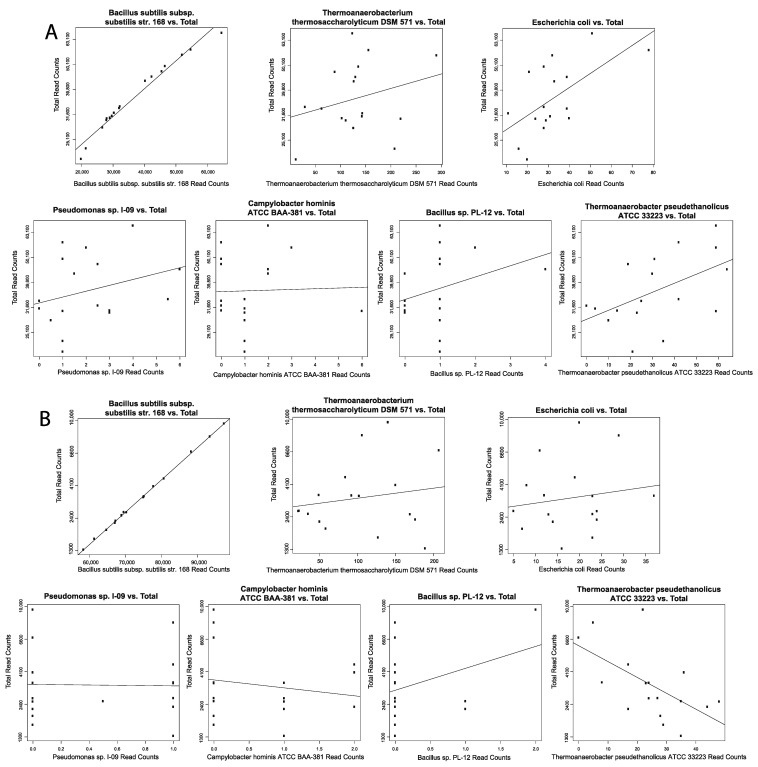
Contamination correction. Spearman’s correlation for select disease severity associated microbes. Scatterplots showing microbe abundance vs. total microbial reads for the most significantly differentially abundant microbes from (**A**) PBMC COVID-19 samples and (**B**) PBMC normal samples.

Scatter plots using Spearman’s correlation to correlate individual microbe abundance to total microbe reads in each patient. Normal and COVID-19 samples were correlated separately. Scatter plots that display a vertical or near vertical regression line deemed the microbe a contaminant, and no GSEA and immune cell correlated microbes were found to be contaminants (Figure 7). Scatter plots for BALF COVID-19 samples (Figure S1A), BALF normal samples (Figure S1B), lung biopsy COVID-19 samples (Figure S1C), and lung biopsy normal samples (Figure S1D).

## 4. Discussion

Few studies have investigated the lung microbiome in COVID-19 patients, and no studies to date have investigated the blood microbiome in COVID-19 patients. In this study, we identified individual bacterial and fungal sequences via high-throughput RNA sequencing differentially abundant between COVID-19 and normal patients. Despite no explicit contamination correction for all microbes identified, we are confident that our findings of immune cell abundance and GSEA pathway correlated dysregulated microbes are authentic due to no contaminants found through our Spearman’s Correlation results.

We identified 91 bacteria and 14 fungus differentially abundant in lung biopsy, 13 bacteria and 9 fungus differentially abundant in PBMC, and 12 bacteria and 57 fungus differentially abundant in BALF. We demonstrate significant associations between many bacterial and fungal species from the lungs and blood and COVID-19, suggesting that the lung and blood microbiota could play a role in modulating host immune response and potentially influence disease severity and outcomes. Specifically, the depletion or enrichment of above listed key bacterial and fungal species in COVID-19 patient samples were associated with downregulation of the anti-inflammatory IL-4 signaling and associated with upregulation of IL-2, IL-3, IL-5, IL-6, IL-7, IL-10, IL-20, IL-22 and IL-23 signaling, IFNα signaling pathways, TGFβ signaling pathways, inflammatory pathways, and immune dysregulation. As shown in a previous study, “shifts in cytokine profiles mediated by changes in the microbiota may also promote epithelial injury and fibrotic outcomes” in chronic lung disease and in COVID-19, and similar associations were observed in our study [24,25].

Across lung biopsy samples, *Campylobacter ureolyticus*, *fungal* sp. *JF54*, *Ochrobactrum anthropi*, and *uncultured beta proteobacterium* correlated to upregulation of TCR activation, IFNα signaling, activation of the complement system, tumor necrosis factor signaling, and several inflammatory interleukin signaling pathways. Burgos-Portugal et al. demonstrated that IFNα causes cells to produce significantly greater amounts of IL-8 and thus inflammation, and cells infected with *Campylobacter ureolyticus* produced even higher levels of IL-8 and thus more inflammation [26]. *Ochrobactrum anthropi* is emerging as an opportunistic pathogen that causes infections in severely ill or immunocompromised patients [27] and has been reported as the cause of diverse inflammatory ailments including infective endocarditis [28,29], endophthalmitis [30], meningitis [31], and osteomyelitis [32]. This previous research lends support to our findings that these microbes may be implicated in modulating the immune response to COVID-19 producing a pro-inflammatory environment and thus worse outcomes for COVID-19 patients. *Fungal* sp. *JF54* and *uncultured beta proteobacterium*, while not thoroughly studied before, had similar associations as *Ochrobactrum anthropi* and *Campylobacter ureolyticus*. *Ochrobactrum anthropi* and *uncultured beta proteobacterium* additionally correlated to decreased gamma delta T cell abundance. Gamma delta T cells play an important role in immunosurveillance in mucosal and epithelial barriers in the lungs and have been demonstrated to play a critical protective role in response to both SARS-CoV-1 and SARS-CoV-2 [33]. This may be another potential mechanism by which opportunistically pathogenic microbiota modulate the response to COVID-19, by disrupting the balance between a sufficient antiviral and hyper-inflammatory response by decreasing the appropriate antiviral function of gamma delta T cells. Furthermore, *Streptococcus sanguinis* has previously been found to be opportunistically pathogenic when the host already suffers certain inflammatory ailments [34]. This lends support to our findings that *Streptococcus sanguinis SK1 = NCTC 7863* may influence the immune response to COVID-19 by upregulating inflammatory IL-6 signaling and downregulating IL-4 signaling. Interestingly, all other differentially abundant *Streptococcus* species did not have significant correlations to inflammatory pathways, which does not support the findings of other studies that have demonstrated *Streptococcus* species correlating to COVID-19 severity [35].

Across BALF samples, *Saccharomyces cerevisiae YJM1444*, *Syncephalastrum monosporum* var. *Pluriproliferum*, *Candida parapsilosis*, and *Histoplasma capsulatum* were correlated to upregulation of TGFβ signaling pathways and inflammatory interleukin signaling pathways. These four microbes also correlated to increased M1 macrophage abundance. Incidentally, other microbes had similar associations but have been previously shown to be only pathogenic to plants. It has previously been shown that in severe COVID-19, SARS-CoV-2 triggers a chronic immune reaction instructed by TGFβ, which contributes to chronic upregulation of IL-6 signaling and B cell activation leading to worse COVID-19 outcomes [36]. By modulating inflammatory interleukin pathways and TGFβ signaling pathways, the above microbes may promote a pro-inflammatory response to COVID-19 worsening patient outcomes. *Syncephalastrum monosporum* var. *Pluriproliferum* also correlated to the local acute inflammatory response pathway. These particular species of *Syncephalastrum* and *Saccharomyces* have not been thoroughly studied, other species have been found implicated in other pulmonary ailments and inflammation in immunocompromised patients and are emerging as opportunistic pathogens. Histoplasma capsulatum and *Candida parapsilosis* are also emerging as opportunistic pathogens, as demonstrated in past studies [37,38,39,40]. The above four microbes in addition to *Tremella mesenterica DSM 1558* correlated to upregulation of RIG-I-like receptors, and RIG-I-like receptors detect viral RNA and mediate the host antiviral response by inducing IFN 1 transcription [41]. Additionally, these microbes may contribute to a hyperinflammatory response by overactivation of the inflammatory M1 macrophages and fewer macrophages differentiating to the anti-inflammatory M2 macrophage mediated by upregulation of inflammatory cytokine signaling and downregulation of IL-4 signaling, as excessive M1-polarized immune responses has been shown to lead to tissue damage in inflammatory diseases [42]. Interestingly, *Fungal* sp. *57* was found inversely correlated with local acute inflammatory pathways and inflammatory interleukin signaling and may play a beneficial role in immunomodulation in COVID-19. Previous study has found that SARS-CoV-2 allows anaerobic bacteria to colonize the lungs and consequently disrupt lung homeostasis [43]. We found four *Haemophilus influenzae* species, two *Bacteroides* species, and *Chlorobium phaeobacteroides BS1*, all being anaerobic bacteria, to have greater abundance in COVID-19 samples in BALF tissue; however, the majority of our data does not corroborate the previous finding that anaerobic bacteria colonize the COVID-19 effected lung, as we found that the majority of bacterial abundance in both lung biopsy tissues and BALF tissues are facultative anaerobic bacteria in both normal samples and COVID-19 samples, and the anaerobic species *Veillonella parvula DSM 2008* and *Haemophilus parainfluenzae T3T1* abundance was greater in normal samples in lung biopsy tissue. Other studies characterizing the COVID-19 respiratory tract microbiomes have found that *Streptococcus* and *Veillonella* dominate the upper respiratory tract and *Streptococcus* and *Haemophilus* abundance are the most important features for segregating COVID-19 clinical outcomes [35,44]. Our findings do not corroborate that *Streptococcus* and *Haemophilus* are most associated with COVID-19 severity nor do *Streptococcus* or *Haemophilus* dominate the lung microbiome in our study. This may provide evidence of some difference between the upper respiratory tract microbiome and the lung microbiome or that the particular identity of commensal microbes populating the lung microbiome may be less important than the severity of differential abundance of commensal microbes. This conclusion may be supported by other studies finding decreased microbiota alpha-diversity in the lung of COVID-19 patients [45]. Our study does corroborate the finding of dysbiosis of commensal microbes and increased abundance of opportunistic pathogens.

Across PBMC samples, *Campylobacter hominis ATCC BAA-381* and *Thermoanaerobacterium thermosaccharolyticum DSM 571* both correlated to upregulation of retinol metabolism and retinoic acid biosynthesis, which both play key roles in regulating antiviral immune response [22,23]. Dysregulation in metabolism and transport of retinol may contribute to inflammatory responses to infection. Furthermore, this dysregulation of retinol metabolism and retinoic acid biosynthesis may play a role in the upregulation of RIG-I-like receptors that was observed in the BALF samples, as retinoids induce RIG-I expression in concert with IFN signaling to lead to the secretion of various pro-inflammatory cytokines [46]. Bacillus sp. PL-12 abundance correlated to decreased plasma cell abundance and correlated to upregulation of IFNα signaling, inflammatory interleukin signaling, and TGFβ signaling, whereas *Bacillus subtilis* subsp. *subtilis* str. *168* inversely correlated to IFNα signaling and inflammatory interleukin signaling, which may explain their correlations to disease severity. Additionally, *Bacillus subtilis* subsp. *subtilis* str. *168* correlated to increased plasma cell abundance and memory B cell abundance and microbes correlated with COVID-19 severity, namely *E. coli*, *Pseudomonas* sp. *I-09*, and *Bacillus* sp. *PL-12* correlated to decreased plasma cell abundance and memory B cell abundance. Previous study has found an association between plasma cells in the blood in patients with severe COVID-19 and improved survival [47]. In corroboration with all of our methods and correlations involving *Bacillus subtilis* subsp. *subtilis* str. *168*, in addition to the strength and linearity of correlation in our Spearman’s Correlation results make us believe future study of this microbe in particular may potentially be useful in immunotherapy for treating COVID-19. *Campylobacter hominis ATCC BAA-381* and *Pseudomonas* sp. *I-09* have not been previously thoroughly studied; however other species of *Campylobacter hominis* and other *Pseudomonas* have been found to induce inflammation in other diseases such as gastroenteritis lending support to our findings that they may modulate the host immune response to COVID-19 [48,49,50,51]. However, it remains unknown if the above inflammatory-associated lung and blood bacterial and fungal species enriched in COVID-19 do in fact play an active role in the prognosis of COVID-19 or simply flourish opportunistically due to a depletion of other lung and blood microbes, but our correlations to immune dysregulation suggest they do in fact play a role in the prognosis of COVID-19.

Our study has several limitations. First, our study used a fairly small sample size for each sample type with 17 PBMC COVID-19 samples and 17 PBMC normal samples, 12 BALF COVID-19 samples and 20 BALF normal samples, and 8 lung biopsy COVID-19 samples and 8 lung normal samples. While enough samples were used to identify numerous dysregulated microbes, further studies with greater sample sizes could be used to increase statistical power and confirm our results. Secondly, metadata of age and disease severity were only available for the PBMC samples used. Further studies correlating microbe abundance to COVID-19 severity using BALF samples and/or lung biopsy samples to identify microbes of the lung microbiome directly correlated with COVID-19 severity should be performed. Thirdly, our study used samples sourced from three different hospitals, and as the human microbiome is highly impacted by geography, our findings may not necessarily reflect the lung and blood microbiome of COVID-19 patients from different geographies. Regardless of these limitations, our study demonstrating associations between microbes found in the lung and blood to immune dysregulation pathways and our associations between microbes in the blood to COVID-19 severity suggests that the lung and blood microbiome are likely implicated in modulating host inflammatory responses to COVID-19 and thus COVID-19 severity.

## Data Availability

RNA sequencing data for PBMC samples are available at Gene Expression Omnibus (accession code GSE152418). RNA sequencing data for lung biopsy samples are available at Gene Expression Omnibus (accession code GSE147507). RNA sequencing data for BALF samples are available at Genome Sequence Archive (accession code HRA000143 and CRA002390).

## Supplementary Materials

The following are available online at https://www.mdpi.com/article/10.3390/cells10061452/s1, Figure S1: The non-contaminant microbes’ plots in lung biopsy and BALF samples. Scatterplots showing microbe abundance vs. total microbial reads for GSEA and immune cell abundance correlated microbes from (A) BALF COVID-19 samples, (B) BALF normal samples, (C) lung biopsy COVID-19 samples, and (D) lung biopsy normal samples.
