# Supplementary material for: Identification of Lung and Blood Microbiota Implicated in COVID-19 Prognosis
Source: Cells. 2021 Jun 10;10(6):1452. doi: 10.3390/cells10061452 (PMC8226556; doi:10.3390/cells10061452)
Supplement: Supplementary file 1 [file cells-10-01452-s001.zip › cells-1237738-supplementary.pdf]

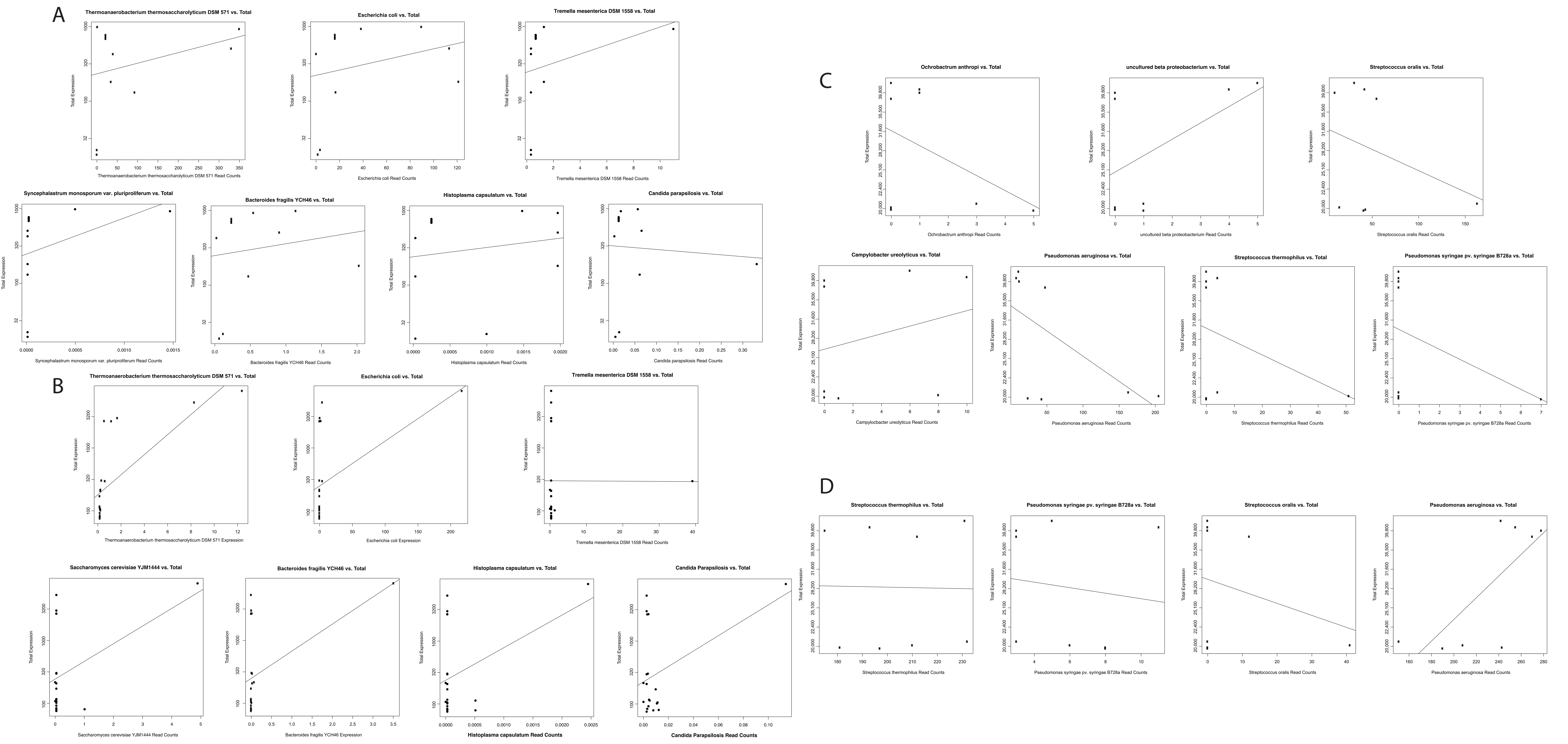
A
Thermoanaerobacterium thermosaccharolyticum DSM 571 vs. Total
Thermoanaerobacterium thermosaccharolyticum DSM 571 Read Counts
Escherichia coli vs. Total
Escherichia coli Read Counts
Tremella mesenterica DSM 1558 vs. Total
Tremella mesenterica DSM 1558 Read Counts
Syncephalastrum monosporum var. pluriproliferum vs. Total
Syncephalastrum monosporum var. pluriproliferum Read Counts
Bacteroides fragilis YCH46 vs. Total
Bacteroides fragilis YCH46 Read Counts
Histoplasma capsulatum vs. Total
Histoplasma capsulatum Read Counts
Candida parapsilosis vs. Total
Candida parapsilosis Read Counts
Total Expression
B
Thermoanaerobacterium thermosaccharolyticum DSM 571 vs. Total
Thermoanaerobacterium thermosaccharolyticum DSM 571 Expression
Escherichia coli vs. Total
Escherichia coli Expression
Tremella mesenterica DSM 1558 vs. Total
Tremella mesenterica DSM 1558 Read Counts
Saccharomyces cerevisiae YJM1444 vs. Total
Saccharomyces cerevisiae YJM1444 Read Counts
Bacteroides fragilis YCH46 vs. Total
Bacteroides fragilis YCH46 Expression
Histoplasma capsulatum vs. Total
Histoplasma capsulatum Read Counts
Candida Parapsilosis vs. Total
Candida Parapsilosis Read Counts
C
Ochrobactrum anthropi vs. Total
Ochrobactrum anthropi Read Counts
uncultured beta proteobacterium vs. Total
uncultured beta proteobacterium Read Counts
Streptococcus oralis vs. Total
Streptococcus oralis Read Counts
Campylobacter ureolyticus vs. Total
Campylocbacter ureolyticus Read Counts
Pseudomonas aeruginosa vs. Total
Pseudomonas aeruginosa Read Counts
Streptococcus thermophilus vs. Total
Streptococcus thermophilus Read Counts
Pseudomonas syringae pv. syringae B728a vs. Total
Pseudomonas syringae pv. syringae B728a Read Counts
D
Streptococcus thermophilus vs. Total
Streptococcus thermophilus Read Counts
Pseudomonas syringae pv. syringae B728a vs. Total
Pseudomonas syringae pv. syringae B728a Read Counts
Streptococcus oralis vs. Total
Streptococcus oralis Read Counts
Pseudomonas aeruginosa vs. Total
Pseudomonas aeruginosa Read Counts

Figure S1: The non-contaminant microbes' plots in lung biopsy and BALF samples. Scatterplots showing microbe abundance vs. total microbial reads for GSEA and immune cell abundance correlated microbes from (A) BALF COVID-19 samples, (B) BALF normal samples, (C) lung biopsy COVID-19 samples, and (D) lung biopsy normal samples.
